# Associative Processing Is Inherent in Scene Perception

**DOI:** 10.1371/journal.pone.0128840

**Published:** 2015-06-12

**Authors:** Elissa M. Aminoff, Michael J. Tarr

**Affiliations:** 1 Department of Psychology, Carnegie Mellon University, Pittsburgh, PA, United States of America; 2 Center for the Neural Basis of Cognition, Carnegie Mellon University, Pittsburgh, PA, United States of America; Centre Hospitalier Universitaire Vaudois and University of Lausanne, SWITZERLAND

## Abstract

How are complex visual entities such as scenes represented in the human brain? More concretely, along what visual and semantic dimensions are scenes encoded in memory? One hypothesis is that global spatial properties provide a basis for categorizing the neural response patterns arising from scenes. In contrast, non-spatial properties, such as single objects, also account for variance in neural responses. The list of critical scene dimensions has continued to grow—sometimes in a contradictory manner—coming to encompass properties such as geometric layout, big/small, crowded/sparse, and three-dimensionality. We demonstrate that these dimensions may be better understood within the more general framework of *associative* properties. That is, across both the perceptual and semantic domains, features of scene representations are related to one another through learned associations. Critically, the components of such associations are consistent with the dimensions that are typically invoked to account for scene understanding and its neural bases. Using fMRI, we show that non-scene stimuli displaying novel associations across identities or locations recruit putatively scene-selective regions of the human brain (the parahippocampal/lingual region, the retrosplenial complex, and the transverse occipital sulcus/occipital place area). Moreover, we find that the voxel-wise neural patterns arising from these associations are significantly correlated with the neural patterns arising from everyday scenes providing critical evidence whether the same encoding principals underlie both types of processing. These neuroimaging results provide evidence for the hypothesis that the neural representation of scenes is better understood within the broader theoretical framework of associative processing. In addition, the results demonstrate a division of labor that arises across scene-selective regions when processing associations and scenes providing better understanding of the functional roles of each region within the cortical network that mediates scene processing.

## Introduction

Scenes are complex stimuli containing rich, statistically regular information about objects, background, context, semantics, and spatial layout at multiple scales. As a core component of scene understanding we extract and learn these regularities, for example, coming to know that certain objects typically appear together or that certain objects are likely to appear in a given spatial relationship [1–3]. Scene categories (e.g., kitchens) emerge as a consequence of these, and other, regular, co-occurring entities (e.g., oven and refrigerator; dishwasher located next to the sink)–something that is reflected in cortical representation [4]. Critically, these associated regularities are not only useful in defining scene categories, but also in predicting which other objects and relations are likely to occur within a scene [5]. More broadly, we hypothesize that many of the mechanisms underlying scene understanding are actually variants of a more general cognitive mechanism: that of *associative processing*. What we mean by *associative* is that the features of any kind of mental representation, irrespective as to whether that representation is nominally visual, linguistic, etc., are related to one another via learned associations. Moreover, these associations are not necessarily modality-specific–for example, visual representations are likely to carry many semantic and affective associations not directly present in the image. At one level, this claim may seem to recapitulate the extant literature on association learning and relational memory [6,7]. However, with respect to scene understanding, we construe contextual associative processing as a more semantically-driven process in which associations are not arbitrary relations, but rather relations that emerge as a consequence of their shared context within the larger scene. That is, associations are not simply frequently co-occurring features pairs; instead they are pairs that both co-occur and add meaning to scenes. Furthermore, the mechanism by which scenes are perceived, recognized, and understood, is through means of processing the associations elicited from components of the scene. Here we investigated, using fMRI, whether different types of relations, all falling under the heading of associative processing, reliably recruit specific components of the network of brain regions known to be scene selective.

More specifically, three regions of the cortex that have been identified as responding selectively to visual scenes: the parahippocampal cortex/lingual region commonly referred to as the “parahippocampal place area” (PPA) [8–12]; the retrosplenial complex (including the retrosplenial cortex, and portions of the posterior cingulate and precuneus, RSC) [13–15]; and a lateral occipital region termed the “occipital place area” (OPA, also referred to as the transverse occipital sulcus, TOS) [16–18]. All three of these brain regions are *functionally* defined by comparing the BOLD signals–as measured by fMRI–arising from viewing scenes to those arising from viewing objects or faces (e.g., [19]).

At the core of neurally-based theories of scene perception are the issues of the computations and representations instantiated in each of these brain regions. For the most part, the literature has focused on more on the latter, characterizing the nature of the information encoded about scenes. For example, it has been suggested that scenes are analyzed in terms of their spatial global properties, such as the ‘spatial envelope’, which takes into account a scene’s overall spatial structure and layout [20,21]. Empirically, it has been demonstrated that these global properties are salient cues for categorizing scenes in both behavior [22] and in patterns of neural activity [23–26]. Indeed, as compared to other factors that characterize scenes (i.e., content and depth), the expanse of the scene (i.e., its spatial boundary) has been found to be the most effective in accounting for variations in the neural responses within scene-selective brain regions [23,24]. More generally, spatial information defining the geometric layout of a scene has often been implicated in determining the neural responses arising from scene-selective brain regions [27].

In contrast, non-spatial properties can account for variations in the neural responses associated with scene processing and representation. For example, Harel et al. [11], found that the neural encoding of scenes captures information about single objects (i.e., non-spatial) as well as background spatial properties. Supporting this point, strongly contextualized objects also elicit neural activity within scene-selective cortex [28,29]. Finally, objects present as the contents of a scene can also modulate activity in scene-selective regions. For example, scenes where the most salient object carries strong contextual associations (e.g., a scene with a parking meter) recruit scene-selective regions more than do scenes with a salient object carrying weak contextual associations (e.g., a scene with a squirrel) [30]. Overall, scene-selective brain regions are encoding a rich collection of information, including global and local properties, spatial background information, and individual object tokens.

One concern with the current state of the field is that the list of critical properties keeps growing (e.g., big/small, crowded/sparse, three-dimensionality [31–33]), with some theories seemingly contradicting others about which properties are critical in scene understanding. Here and elsewhere, we posit that many dimensions of scene understanding may be better understood within a more general framework in which scenes are encoded with respect to their associative properties [28,30,34,35].

Critically, associative properties are not restricted to the spatial or the global domain, and can account for why information regarding spatial layout and single objects play an important role in scene understanding and concomitant neural responses. Associative processing not only offers an explanation for what particular kinds of information are present in scene representation (e.g., objects and spatial information), but also a basis for explaining how and why this information is important. For instance, on average, spatial layout and global properties may carry more associative information, and thus are typically prominent in accounts of the mechanisms mediating scene recognition. Consider *expanse*, a salient, organizing principle within scene representations: expanse may be construed as a superordinate category strongly associated with many other properties of a scene, for instance nature, navigating, hiking, and, therefore, is likely to be strongly predictive of additional scene information.

Strikingly, a recent study by Mégevand and colleagues supports the hypothesis that associative processing is fundamental to scene understanding. Mégevand et al. [36] found that stimulation of the human PPA region using intracranial EEG produced a visual hallucination of not just spatial layout, but a complete change of context: under stimulation, the patient experienced a shift from the actual hospital setting to the perception of an Italian pizza shop that included hallucinations of individuals and objects that were consistent with the overall hallucinated context (i.e., the people looked Italian and were wearing aprons).

To test these ideas, in particular, that associative processing is inherent in scene understanding, we explore whether the network of brain regions recruited in scene perception is also recruited when processing non-scene-like stimuli that contain associative information. The critical point being that if these regions are active when processing the associations learned between non-scene-like novel stimuli than these regions must not be involved in only processing the visual properties of the scene, but rather, are processing what is associated with the components of the scene as learned from previous experience. Here we focus on the associative processing of concurrent object identities (i.e., semantic associations) and spatial relations between objects (i.e., spatial associations)–of course, other domains of associative processing, for example, diagnostic mid-level features, almost surely play some role as well. For both semantic and spatial associations, we predict that the three typically-identified scene-selective brain areas, the PPA, RSC, and OPA, will all exhibit selective activity for both scenes *and* for associations in general, without necessarily having any obvious “scene-like” qualities.

More generally, we are positing that scene processing is best understood as, at least in part, arising from a more general mechanism–that of associative processing. The fact that this mechanism is more general does not make it vague. Our point is that learning and encoding relations between tokens (“associations”) is a fundamental process across many cognitive domains and, critically, this process has explanatory validity for scene processing. As such, it is part of the field’s efforts to elucidate the functional basis of scene perception rather than simply presenting an account in which the explanation is scene processing *qua* scene processing. Reframing scene processing as involving a more general mechanism helps articulate the means by which scene perception occurs.

**Fig 1:**
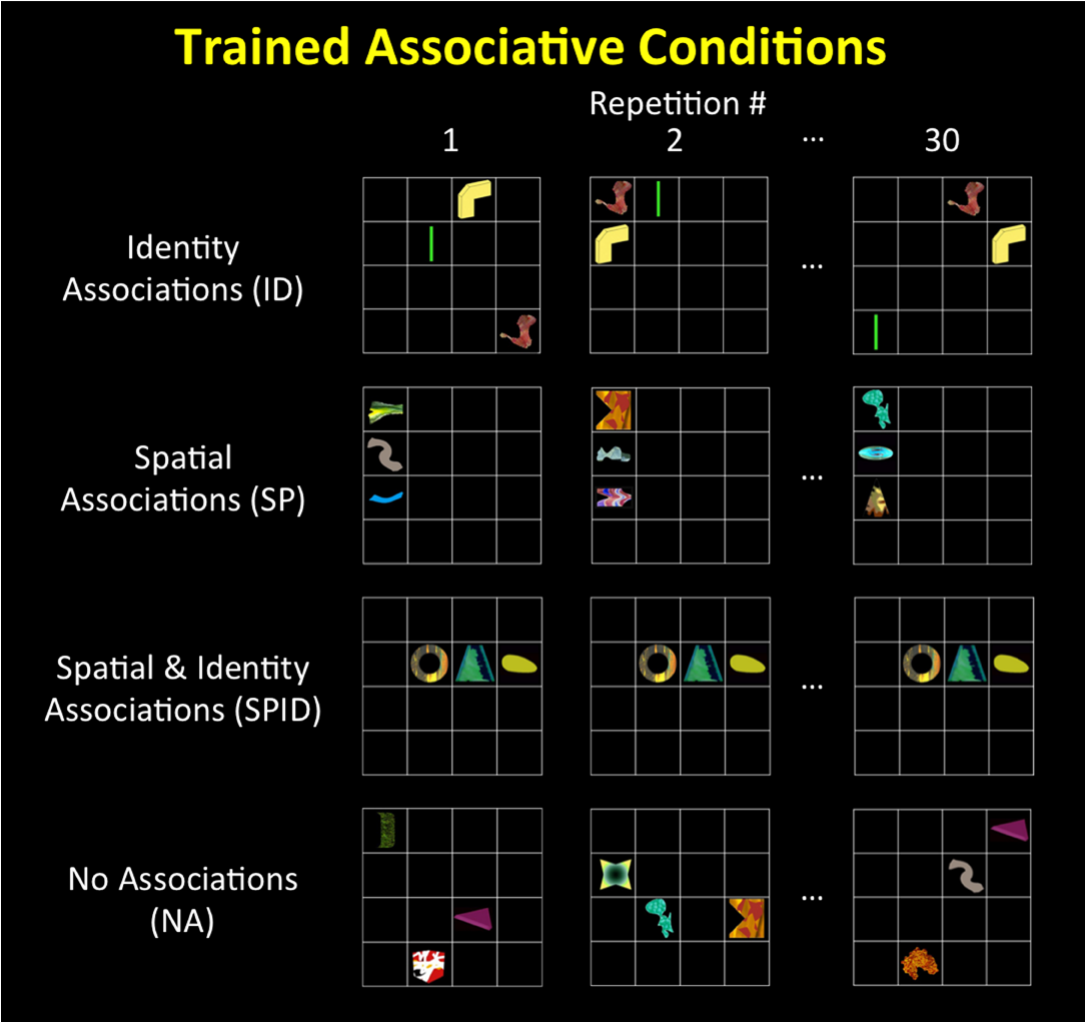
Experimental Conditions. Examples of the training phase. *Identity* (ID)–the same three shapes presented together in random configurations; *Spatial* (SP)–three random shapes presented in the same configuration; *Spatial-Identity* (SPID)–the same three shapes always presented together in the same configuration; *No Association* (NA)–three random shapes presented in random configurations. Each stimulus was repeated thirty times in the training phase.

To test these predictions, participants were scanned using fMRI while they engaged in processing either everyday scenes or associations between meaningless shapes. For the latter condition, participants, immediately prior to the scanning session, performed a task (unrelated to the associations) in which they implicitly learned spatial or identity associations between configurations of novel shapes (Fig 1). First, to compare brain activity from learning novel associations to the processing of scenes, we correlated the distributed, voxel-by-voxel pattern of activities arising from these two conditions, thereby testing whether similar neural mechanisms mediate both general associative processing and scene perception. Second, consistent with previous work ([28,31,34,37]) we asked whether the neural encoding of scenes shifts across association types–from non-spatial or semantic scene properties to spatial scene properties–as one moves from the anterior to the posterior PPA. Together, these analyses shed light on the hypothesis that a single underlying principle, associative processing, accounts for how scenes are represented in the brain.

Although prior studies have manipulated spatial and identity associations by having participants learn meaningless patterns of shapes [34], ours is the first study to directly compare such associative processing with the processing of everyday scenes. Moreover, we include two new baselines to better understand whereby effects of associations arise: weak contextual objects and scrambled images. Finally, we are careful to collect an additional independent dataset to functionally define scene-selective regions. These new conditions are all advances–in terms of explicating the processing of scene-selection regions–relative to our earlier study in which we used the same shapes and trained participants over a two-week period [34]. At the same time, we also include several methodological changes: training was much briefer; the task was changed from an explicit task to an implicit task; a spatial-only associative condition was included to isolate spatial processing independently of identity associations; and the no-association condition provides a better control in that it now shows the same number of shapes as the association condition (i.e., 3). From a cognitive neuroscience perspective, we also have included additional regions of interest, focusing not only on the PPA, but also the RSC and the OPA–both scene-selective regions. Lastly, advances in analysis methods allow us to apply a multi-voxel approach (“MVPA”) correlating the *pattern* of activity across voxels within a given region across conditions of interest. This analysis addresses not just what these regions are doing on average, but also how these associations support scene representation.

## Results

To investigate the role of both spatial and non-spatial associations in neural scene encoding, we trained participants to recognize novel spatial (SP) associations, non-spatial (ID) associations, and joint spatial and non-spatial (SPID) associations between novel visual tokens (Fig 1). Training of the associations was done implicitly to control the extent to which various strategies or assignments of verbal labels were employed. Participants successfully learned these associations as confirmed by the explicit test post training (chance = 33%, average performance 58%, *t*(14) = 4.13, *p* < .001). Following this, we used fMRI to measure the BOLD activity associated with the processing of these trained associations, real-world scenes, objects with weak contextual associations, and scrambled pictures. To focus on how such spatial and non-spatial associations are encoded, we compared each association condition (SPID, SP, ID) to the control condition (NA). To ensure that any differences in BOLD responses in these comparisons were attributable to the learned associations, the visual descriptors and participant familiarity with the stimuli were equated between the association and control conditions. To examine the relationship between the neural regions mediating associative processing and those mediating real-world scene understanding, we compared scenes with objects and scrambled pictures. We hypothesized that associative processing and real-world scene understanding would elicit similar patterns of BOLD activity if both visual processing tasks are supported by similar–at least in part–psychological and neural mechanisms.

To examine these predictions in the context of the neural network believed to support scene processing, we used an independent functional localizer to define scene-selective brain regions within individual participants. This localizer yields the functionally-defined regions typically associated scene processing: the PPA, RSC, and OPA.

### fMRI: Whole brain results

**Fig 2:**
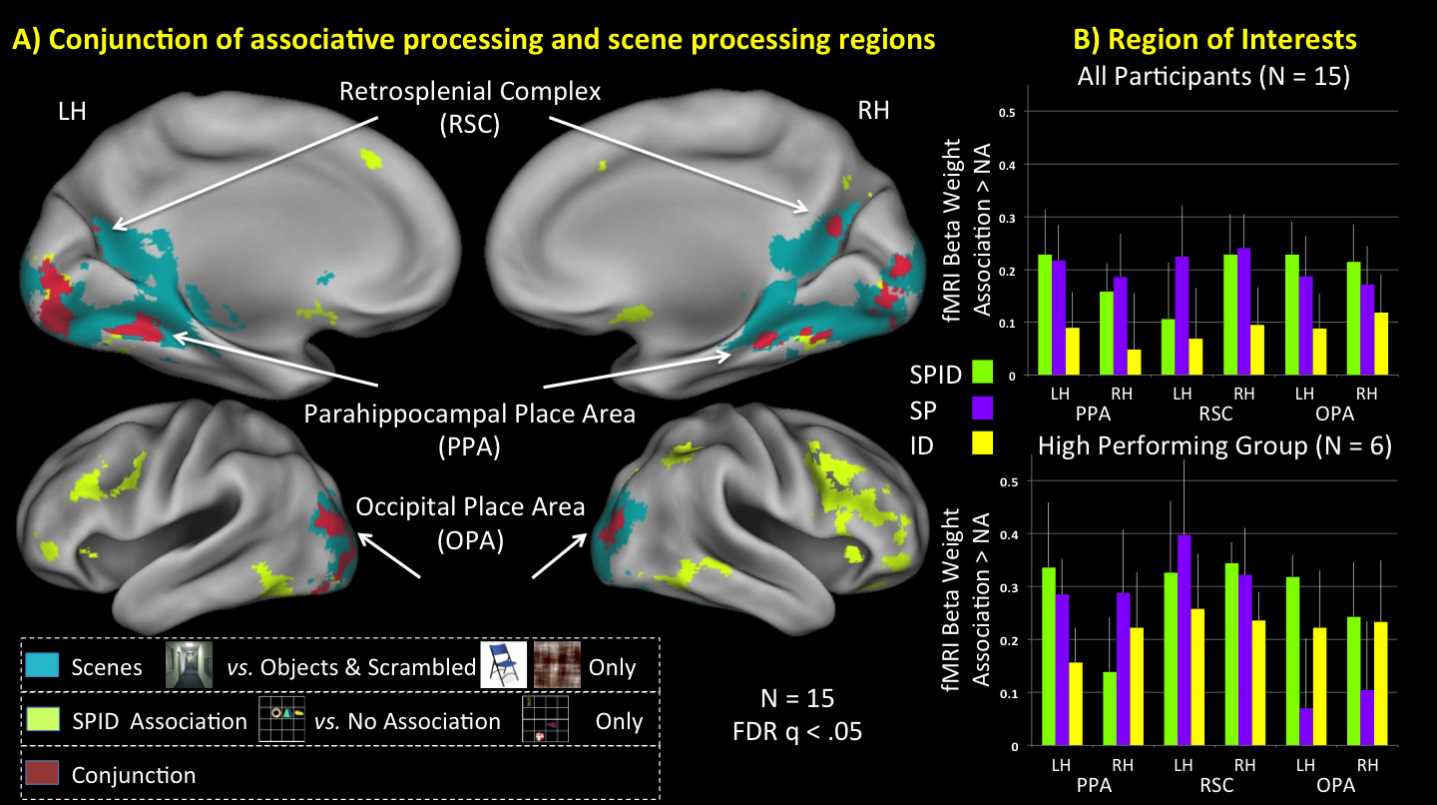
A) Whole brain analysis comparing BOLD activity elicited for the associative shapes (SPID vs. NA, neon green) with the activity elicited for the scenes (Scenes vs. Objects and Scrambled, teal). Both contrasts revealed regions of the brain with overlapping significant differential activity and particularly within the PPA, RSC, and OPA. B) Region of interest analysis for the PPA, RSC, and OPA. Bar graphs show the activity that was greater the control NA condition; negative values would indicate that NA > associative conditions, which was not found. B–bottom) a subset of the participants who performed above chance in all three associative conditions.

The SPID condition was designed to be the closest in structure to a real world scene in that it contains associations both in content (i.e., three shapes; identity associations) and in spatial arrangement (i.e., a fixed configuration; spatial associations). As such, we predicted that whole brain activity elicited for the SPID versus the NA condition would reveal responses within “scene-selective” regions, which, in fact, is what was observed in our data (Fig 2A and S1 Table). Fig 2 shows the overlap of activity related to the processing of associative shapes (SPID vs. NA) and the activity related to the processing of scenes (Scenes vs. Objects & Scrambled) for all three scene-selective regions: this overlap was observed bilaterally within the PPA and the OPA; within the RSC this overlap was observed in the right hemisphere (RH), with a minimal region in the left hemisphere (LH). This overlapping significant differential BOLD activity in the PPA, RSC, and OPA supports the hypothesis that the computations within scene-selective regions are not specific to the processing of scenes *per se*, but more generally related to the processing of associations. Finally, in addition to observing overlapping activity within the three main “scene-selective” regions, we also observed overlap within fusiform gyrus, inferior temporal gyrus, early visual regions in the calcarine sulcus, and the cerebellum (S1 Table).

Despite this overlap, there were also differences between the activity arising from the SPID/NA and scenes/objects contrasts. In particular, the activity related to scene processing was far more spatially extensive–a pattern that may be accounted for by the fact scene stimuli have many associations, whereas the novel shape stimuli have a minimal number. At the same time, several brain regions were uniquely related to the SPID/NA contrast, including the lateral prefrontal cortex, lateral occipital cortex, insula, dorsal anterior cingulate, and right hemisphere superior parietal cortex. Some of this activity, especially within the prefrontal cortex, may be accounted for by the continued learning of the stimuli during the fMRI phase of the experiment [38,39].

### fMRI ROI analysis: average signal

The mean beta weights for each of the trained conditions (SPID, SP, ID, NA) were extracted for each region of interest (PPA, RSC, OPA) in each hemisphere to examine the overall effect of associative processing within the scene-selective network (Fig 2B). To identify effects of associations across these different types of associations, an ANOVA across these four conditions was run for each ROI. A main effect of Association was significant in the LH PPA (*F*(3,42) = 5.13, *p* < .004), RH RSC (*F*(3,42) = 6.0, *p* < .002), and both the LH and RH OPA (*F*(3,42) = 3.58, *p* < .021; *F*(3,42) = 3.33, *p* < .03). A main effect was found to be marginally significant in the RH PPA (*F(*3,42) = 2,16, *p* < .11) and the LH RSC (*F*(3,42) = 1.95, *p* < .14). Planned comparisons demonstrated that there was a significant increase in BOLD activity for the associative conditions over the No-Associative condition: SP versus NA was significant in all six ROIs (*p*’s < .04), and SPID versus NA was significant in all ROIs (*p*’s < .02) except for the LH RSC. Activity in the ID versus NA was always numerically higher, however this comparison did not reach significance in any of the planned comparisons (*p*’s > .12).

One question is whether there is a relationship between the BOLD activity associated with spatial and identity processing and how well the participants learned these associations. To investigate this possibility, the mean beta weights from each ROI were correlated with each participant’s mean performance across two tests of association learning–one administered following training and one administered following MRI scanning. In both the LH and RH RSC there was a significant correlation between how well participants learned SPID associations and the SPID/NA neural contrast (LH: *r*(15) = .55, *p* < .03; RH: *r*(15) = .57, *p* < .02). Surprisingly, we also observed negative correlations with learning: there was a negative correlation between how well participants learned SP associations and the SP/NA neural contrast in both the LH and RH OPA (LH: *r*(15) = -.61, *p* < .02; RH: *r*(15) = -.62, *p* < .015) and in the RH PPA (*r*(15) = -.55, *p* < .03; however, one participant seemed to be driving this effect, and when removed, the correlation was no longer significant).

To obtain a clearer qualitative snapshot of these effects, we divided participants into two groups: those that showed above chance learning in each condition (N = 6), and those that did not (N = 9). Consistent with the correlations reported above, there was a significant effect of Group in the LH RSC (*F*(1,13) = 5.01, *p* < .04) and a marginal effect in the RH RSC (*F*(1,13) = 3.97, *p* < .07). There was also a Group x Association interaction for the LH OPA (*F*(3,39) = 3.25, *p* < .03); likely driven by performance in the SP condition. An ANOVA using only the above-chance group and the associative conditions as factors revealed main effects of Association in the RH RSC and the LH OPA consistent with the results obtained using all participants (RH RSC: *F*(3,15) = 9.4, *p* < .001; *F*(3,15) = 3.69, *p* < .04). However, in the LH RSC, an analysis of the above-chance group now reveals a significant effect of Association (*F*(3,15) = 3.37, *p* < .05). For planned comparisons, there was no significant effect of SP versus NA in the LH OPA, and both the LH and RH RSC showed significant effects for the ID versus NA (LH: *p* < .05; RH: *p* < .001).

### fMRI ROI analysis: distributed pattern

As discussed above, the results of our study–considered in terms of average signal from each ROI–suggest that the scene-selective brain regions also appear to be recruited by (visual) associative processing. To examine the relationship between scene and associative processing in more detail, we also explored the similarity between the distributed patterns of voxel activity across the association and scene conditions. In particular, variance across voxels may carry meaningful information with respect to the degree to which two spatially overlapping neural processes share common computational underpinnings. In our study, high similarity between the neural patterns arising from the association and the scene conditions would provide further evidence that these two tasks are based on similar neural representations and/or computational mechanisms. In contrast, low similarity might suggest differences in the underlying mechanisms mediating the encoding of these two kinds of visual information (albeit within the same neural “neighborhood”). To investigate this issue, we extracted unthresholded *t* values for the contrasts scenes versus baseline and associative shapes (SPID, SP, and ID collapsed) versus NA from each scene-selective ROI and cross-correlated these *t* values on a voxel-by-voxel basis for each participant. All *r* values were then fisher-corrected and tested for significance against zero. *t*-tests demonstrated that these correlations were significant in all scene-selective ROIs except for the LH RSC, where the correlation was only marginally significant (LH PPA: $\bar{r}$ = .17, *t*(14) = 3.05, *p* < .009; RH PPA: $\bar{r}$ = .15, *t*(14) = 3.06, *p* < .008; LH RSC: $\bar{r}$ = .08, *t*(14) = 1.88, *p* < .08; RH RSC: $\bar{r}$ = .15, *t*(14) = 4.29, *p* < .001; LH OPA: $\bar{r}$ = .23, *t*(14) = 7.38, *p* < .000003; RH OPA: $\bar{r}$ = .16, *t*(14) = 2.24, *p* < .04).

Given the reliable correlation between the patterns of activity elicited for associative processing and for scene processing, as a control, we examined whether other, non-associative, visual tasks would elicit similar patterns of activity in these ROIs. More specifically, we correlated contrasts between weak contextual objects versus baseline and between scenes versus baseline with contrasts arising from each of the three association conditions (SPID vs. NA, SP vs. NA, and ID vs. NA). These correlations were used in a 2 x 3 ANOVA (2 stimulus types x 3 types of associations) that revealed significant effects of stimulus type in four of the six ROIs (LH PPA: *F*(1,14) = 4.38, *p* < .055; RH PPA *F*(1,14) = 8.88, *p* < .01; RH RSC: *F*(1,14) = 8.21, *p* < .01; RH OPA: *F*(1,14) = 5.29, *p* < .04), a marginal effect in the LH OPA (*F*(1,14) = 3.69, *p* < .08), and no effect in the LH RSC (*F*(1,14) = 1.46, *n*.*s*.). That is, the distributed pattern of activity across scene-selective ROIs shows significant correlations for the patterns elicited by associative processing and scene understanding. Critically, this effect was over and above that elicited by object processing, in that the same relationship was not observed for weak contextual objects. As such, these results are consistent with and reinforce our claim that the same mechanisms mediate both associative processing and scene understanding.

### Anterior to posterior processing in the PPA

Although the general principle of associative processing may apply broadly across scene-selective ROIs, there is some evidence that within the parahippocampal region, anterior and posterior regions may support different kinds of associations [34,37,40,41]. In particular, we posit that anterior regions process non-spatial associations such as those realized in the ID condition, whereas posterior regions process spatial associations such as those realized in the SP condition. We tested this hypothesis by examining two contrasts within scene-selective parahippocampal regions: SP versus NA and ID versus NA. To identify the appropriate scene-selective ROIs for this analysis, we used the scenes versus baseline contrast to capture all scene-related activity within parahippocampal cortex. We then anatomically mapped this region within individuals before separating it into four different subregions along the anterior-posterior axis (see Methods). Within these subregions, only those voxels that demonstrated significant scene-related activity from the scenes versus objects/scrambled localizer were analyzed. However, observed scene-related activity within the parahippocampal/lingual region extended beyond the posterior border of the parahippocampal cortex and into the lingual gyrus. To capture these responses, we also included these more posterior voxels as the most posterior subregion, giving us a total of five subregions covering the PPA from anterior to posterior regions.

Data were analyzed examining both the average activity within each subregion, and the distributed pattern of activity in each subregion correlated with the pattern for scenes. The pattern of average activity across subregions supports a division of labor in which anterior subregions showed the largest differences in the ID versus NA contrast relative to the SP versus NA contrast, while posterior subregions showed the largest differences in the SP versus NA contrast relative to the ID versus NA contrast. In the LH PPA this was significant in both in the interaction of Association by Subregion (*F*(4,56) = 5.85, *p* < .001) and critically, in the linear interaction contrast (*F*(1,14) = 8.05, *p* < .01). The significance of this linear contrast indicates that the transition from identity to spatial associative processing may be construed as a gradient from anterior to posterior regions of parahippocampal cortex. However, the same Association by Subregion interaction was not observed in the RH PPA.

**Fig 3:**
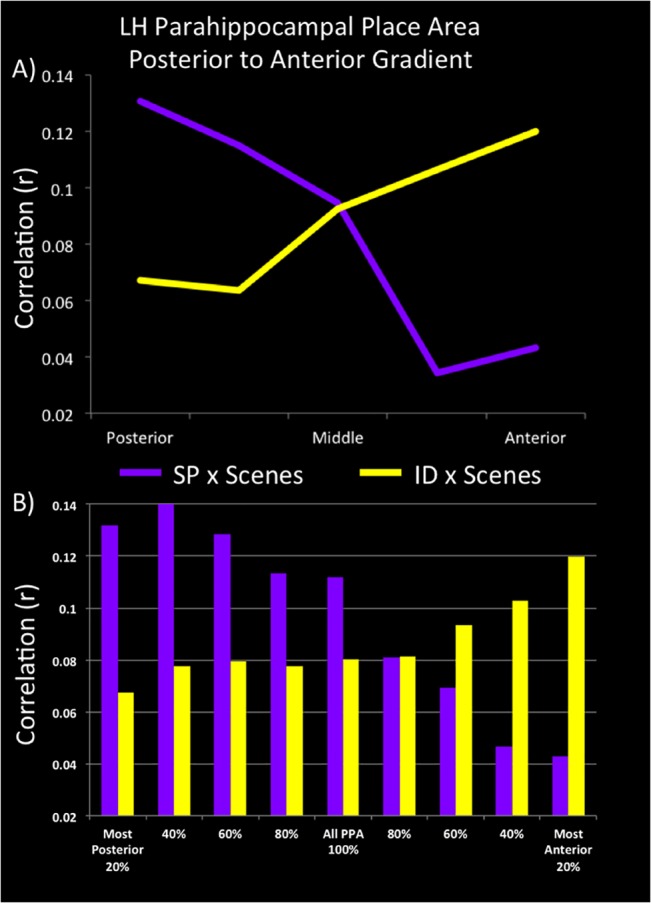
LH PPA posterior (spatial) to anterior (non-spatial) gradient of information processing. A) the correlation in the pattern of activity across voxels in five bins going from the posterior to anterior regions of the PPA for unthresholded t maps of SP vs. NA with Scenes vs. Objects+Scrambled in purple and ID vs. NA with Scenes vs. Objects+Scrambled in yellow. B) Taking the correlation of these contrasts from one extreme end and adding more PPA until the entire PPA is surveyed.

To examine whether identity and spatial associations also capture functionally-relevant properties within scenes, we examined the similarity between the voxel-wise pattern of BOLD responses elicited by identity associations and real-world scenes, and by spatial associations and real-world scenes. We hypothesized that, to the degree that these two types of associations are intrinsic to scene processing, similar patterns of activation across each of the two association conditions and real-world scenes would identify those–potentially separable–neural substrates supporting the representation of these kinds of associations. Unthresholded *t*-values for the ID versus NA and SP versus NA contrasts within each of the five PPA subregions were extracted for each voxel and then cross-correlated within each subregion with the real-world scenes versus objects/scrambled contrast. In LH PPA the interaction within an omnibus ANOVA [the two factors being Subregion (moving from the most posterior to the most anterior) x Association (identity or spatial)] was significant (*F*(4,56) = 2.69, *p* < .04) as well as the critical linear x linear trend analysis (*F*(1,14) = 7.57, *p* < .016) (Fig 3A). Consistent with our analysis of average activity within these subregions, this interaction was not observed in the RH PPA (*F*(1,14) = 1.22, *n*.*s*.) (S1 Fig). However, it is worth noting that within the right hemisphere, the correlation between spatial associations and real-world scenes did exhibit the predicted pattern whereby the strongest correlation was observed in posterior subregions of PPA, with a progressively lower correlation being observed moving in a posterior to anterior direction (S1 Fig).

Overall, our results indicate that the organization of PPA with respect to associative processing is a gradient in which posterior subregions of PPA are biased towards spatial associations and anterior subregions of PPA are biased towards identity associations. Additional support for this claim is shown in Fig 3B, where instead of dividing the PPA into five equal subregions, the correlation was run over progressively more and more of the PPA, starting from the most posterior subregion and adding subregions until the entire PPA was taken into account; similarly, the same analysis is shown starting from the most anterior region progressing to the entire PPA. As illustrated, the most extreme ends of the PPA show the strongest biases towards capturing the similarity of either spatial associations and scenes, or identity associations and scenes; whereas if the whole PPA were to be surveyed, these differences would be masked. Of note, this gradient appears to be truly continuous across posterior and anterior portions of the PPA–even considering only the posterior PPA, we observe a gradient in which the strongest preference for spatial associations is found in the most posterior portions of the posterior PPA. Similarly, considering only the anterior PPA, we observe a gradient in which the strongest preference for identity associations is found in the most anterior portions of the anterior PPA. Finally, an examination of these results on an individual participant basis reveals that this pattern remains highly reliable and, therefore, does not appear to be a by-product of group averaging across slightly offset discrete functional subregions.

## Discussion

Associative processing is inherent in scene understanding, and, importantly, provides an underlying mechanism whereby “scene-selective” brain regions come to be scene selective. Put another way, one of the properties that distinguishes scenes is their rich associational structure. Here we establish that elements of this structure underlie both the how and the why of scene selectivity in human cortex. In particular, we compared the neural responses elicited by simple novel patterns carrying spatial and/or identity associations to the neural responses elicited by real-world scenes, finding that the two classes of stimuli engaged overlapping regions of the cortex, namely the PPA, RSC, and OPA. We take this evidence as support for our hypothesis that associative processing is fundamental to scene understanding, that associations are an important dimension along which scenes are neurally represented, and that scene processing itself is actually a subcomponent of the more general associative processing that occurs across many cognitive domains.

### Associative processing in “scene-selective” cortex

To recapitulate our main result, both univariate and multivariate methods indicate that scene-selective brain regions–the PPA, RSC, and OPA–are likewise engaged when processing both spatial and identity associations using novel stimuli. Particularly compelling is our finding that the voxel-wise, distributed patterns of activity elicited by real-world scene processing and the processing of our novel associative stimuli are similar to one another. This fine-grained similarity suggests that similar computations are involved in processing both types of stimuli, supporting an associative processing model of scene perception. In contrast, many earlier studies explain scene perception using single dimensions, for example, geometrical layout, expanse, navigability, etc. Here we adopt a broader framework in which scene processing is a subset of more general associative and contextual processing. As such, we offer an explanation for scene perception that is multidimensional, encompassing a wide variety of scene properties, including the two investigated in this study–spatial and identity associations.

### Posterior to anterior specialization in the PPA

Building on our general results regarding associative processing, our current findings also establish some specialization (at least in preference) for the processing of associative information across the PPA. In particular, posterior PPA is preferentially recruited by spatial associations while anterior PPA is preferentially recruited by semantic (i.e., non-spatial) associations. To be clear, a functional distinction between anterior and posterior PPA has been reported previously. In particular, this distinction has been based on PPA responses when processing objects associated with spatial contexts (i.e., contexts associated with specific places, for example, an oven and a kitchen) and when processing objects associated with non-spatial contexts (i.e., contexts not tied to specific places, for example, champagne with New Year’s Eve) [28,34,37,40,41]. Here we explicitly investigated this spatial/non-spatial distinction by isolating the two association types in simple, novel stimuli and then relating these controlled stimuli to the processing of real-world scenes containing similar associations. We predicted that this Association-Type x Scene correlation would reveal the expected posterior to anterior progression with spatial properties within scenes selectively recruiting posterior PPA and non-spatial, identity properties within scenes selectively recruiting anterior PPA. Moreover, we expected that this functional division would be continuous, with the similarity in BOLD responses elicited by scenes and trained spatial associations decreasing in a posterior to anterior direction. In contrast, the similarity in BOLD responses elicited by scenes and trained identity associations was predicted to decrease in an anterior to posterior direction. These predictions were confirmed in our study.

This information processing gradient may lie within a broader, more general organization of the parahippocampal gyrus. The gradient of processing identity or non-spatial associative information may continue through the neighboring perirhinal cortex, which is thought to be involved in object and person recognition particularly with regard to combining different features of knowledge, such as a stop sign is red [42]. In contrast, the posterior regions of the PPA border regions where mid-level visual information is processed, which may be more sensitive to spatial information. We do note, however, that this interaction was only observed in the left hemisphere, although the right hemisphere demonstrated a similar gradient of specialization in spatial processing from posterior to anterior. However, the activity related to identity processing was less well organized. This pattern of results is somewhat consistent with the fact that the left hemisphere is often implicated in more semantically-oriented processing, for example, scene categorization, whereas the right hemisphere has been implicated in more spatial processing, for example, as in the visual details between different exemplars of the same category [10]. In *toto*, these patterns hint that the right hemisphere PPA may play a larger role in the processing of the spatial relations within scenes and a lessor role in the processing of scene semantics. In sum, our current functional view of the PPA is as a gradient in which progressively different scene dimensions come to be instantiated.

### The division of labor in the PPA, RSC and OPA

The PPA, RSC, and OPA were all strongly engaged in both associative processing and in scene perception. However, it is unlikely that these three regions play entirely redundant roles in scene understanding–a conclusion supported by the differences we observed between the SP, ID, and SPID conditions. In particular, we suggest that the PPA involves the processing of multiple scene dimensions that reflect different kinds of associative information (e.g., spatial and identity). In contrast, both the RSC and OPA were both more sensitive to identity associations, whether appearing in isolation or in conjunction with spatial associations. Elsewhere we have suggested that the RSC processes the prototypical representation of context, termed a “context frame,” which contains information regarding both the key objects and the spatial relations between them [28,43]. For example a context frame of bathroom would include information regarding a shower, toilet, sink, toothbrush, mirror, as well as spatial information such as the mirror is typically located above the sink. Our present data support this suggestion, indicating that the RSC is involved in both spatial and identity associative processing. Moreover, responses in the RSC were the most predictive of learning: better learning elicited higher neural activity in the RSC, specifically in the conjoined SPID condition. As such, the RSC, as the highest level of associative processing, may be involved in the long-term encoding of contextual associations, that is, the context frame, where both spatial and non-spatial associations are processed. In contrast, the OPA lies in close proximity to the IPS, a region that has anatomical connections with both the ventral stream, potentially conveying identity information, and the dorsal stream, potentially conveying spatial information [44,45]. This provides the anatomical architecture to combine both identity and spatial information within a unified contextual representation. Future studies are needed to explore the specific anatomical connections with the OPA regions, examining whether it is similarly connected to both the dorsal and ventral pathways.

Interestingly, our study also found the neural signal related to the processing of spatial associations from the OPA was negatively correlated with learning. That is, the better the learning of spatial associations, the lower the response of the OPA relative to the control condition. Because the OPA is more posterior than the PPA and RSC, it may be involved in earlier stages of scene processing [16–18]. Our results build on this, suggesting that the initial processing of spatial associations occurs in the OPA, whereas more complex analysis of spatial contextual associations, with increased learning, may occur in, progressively, the PPA and RSC.

## Conclusions

Our results move beyond simply describing which brain regions are selectively recruited by visual scene processing. We invoke associative processing as a computationally-definable construct for predicting the spatial organization of scene-related activity across different scene properties. Consequently, we suggest that the neural mediators of scene processing should not be construed as encapsulated visual modules, but rather as manifestations of associations that reflect the interaction between visual recognition processes and the application of long-term memories arising from past experiences (Aminoff et al., 2013). Within this framework, scene processing is not a purely bottom-up visual process, but rather is an interactive process in which we are constantly considering–in the form of both spatial and non-spatial associations–our past experiences to generate predictions, expectations, and constraints about our physical environment.

More specifically, we suggest that general associative processing mechanisms are sensitive to frequently occurring or repeated relations within scenes, including object identities that co-occur, spatial relations between the objects, and spatial locations for individual objects, all of which facilitate the recognition and categorization of visual scenes. As such, the collection of associations that help to define a scene provides a context for individual elements within that scene. For example, a towel hanging on a rack near a shower would most likely be a bathroom towel, whereas the same towel lying flat on the sand near an umbrella would most likely be a beach towel. We contend that the parsing of these sorts of contextual associations within scenes is fundamental to scene understanding, and that scene understanding construed as such, can then influence cognitive processing in myriad ways.

Within the human brain, we argue that the neural bases for the application of contextual knowledge is a functional gradient spanning different types of associations instantiated across the PPA, and contextually-mediated representations within the RSC and OPA. Most saliently, our observations of selectivity for spatial and identity associations provide compelling evidence that the neural representation of visual scenes is best understood as a consequence of the associative processing in both the spatial and non-spatial domains.

## Methods

Outside the MRI scanner, participants implicitly learned novel identity and spatial associations between heretofore meaningless shapes. Following this training task, participants were explicitly tested as to whether they had learned these associations. Next, participants were scanned using MRI, the functional task being to monitor for a change in fixation color while simultaneously viewing either the trained associative stimuli, everyday scenes, everyday objects, or scrambled images. In addition to these experimental conditions, participants were also run in a scene “localizer” to independently define scene-selective cortical regions [8]. Following MRI scanning, participants were tested to assess how well they had learned the associations. Each experimental session lasted between 2–2.5 hours. All stimuli were presented using Psychophysics Toolbox [46] running under Matlab (Mathworks, Natick, MA) on an Apple Macbook Pro.

### Participants

Fifteen participants (12 females; one left-handed; age range 18–33, mean 24), all with normal, or corrected-to-normal vision, were included in the data analysis–see S1 Supporting Methods for information regarding excluded participants. Written informed consent was obtained from each participant prior to testing in accordance with procedures approved by the Institutional Review Board of Carnegie Mellon University. The Institutional Review Board of Carnegie Mellon University approved all research in the current study. Participants were financially compensated for their time.

### Stimuli

Fig 1 illustrates the four associative conditions, each of which was comprised of novel configurations of colorful, meaningless shapes (as used previously in [34]). Each stimulus image consisted of a 4 x 4 white grid, which subtended a 7.3° visual angle, with three shapes presented in the grid, one shape per grid location–termed “triples”. There were four associative conditions: *Spatial-Identity* (SPID) in which the same three shapes always appeared together in the same locations within the grid; Identity (ID) in which the same three shapes always appeared together in random locations within the grid; *Spatial* (SP) in which the same three positions within the grid were always filled with shapes, but the shapes within the positions were random; and *No-Association* (NA) in which three randomly selected shapes were presented within three random positions within the grid. A total of forty-eight shapes were used (three shapes per a triple): twelve shapes in SPID; twelve shapes in ID; and a pool of twenty-four shapes used across SP and NA. The assignment of shapes to conditions were counterbalanced across participants. The positions used in the SPID and SP conditions consisted of configurations with three adjacent positions, either vertical or horizontal, balanced across participants. The ID and NA conditions consisted of configurations chosen randomly from a pool of 280 possible shape arrangements, none of which included three vertically or horizontally adjacent shapes.

Other images used in the experimental conditions included color pictures of everyday scenes, everyday objects, and phase-scrambled images. Scene images fell into three categories with four exemplars each: hallways, roads, and intersections. Object images were all objects with weak-contextual associations [28] shown on a white background: a folding chair, clock, fan, and garbage can. Images were presented at a 7.3° visual angle.

To localize scene-selective brain regions, a separate scene “localizer” was run that included 84 pictures of both indoor and outdoor scenes and 84 pictures of objects with weak contextual associations shown on a gray background (e.g., a phone). Images were presented at a 5.5° visual angle.

All images were presented against a black background.

### Procedure

Training. Participants were informed that they would see a grid containing three novel shapes and that they were to mentally segment the grid into four quadrants, pressing a button indicating how many of the quadrants contained at least one shape (i.e., 1, 2, or 3). Participants were not provided with any additional instructions, and thus, were unaware that this was a training session or that any of the stimuli would be repeated. Each trial began with a fixation cross (“+”) presented in the middle of the screen for 250ms, followed by a blank screen for 250ms, followed by the grid showing the shape triple for 2500ms. Each triple was presented thirty times during the course of the training phase, for a total of 480 trials (4 triples x 4 conditions x 30 repetitions) over a period of roughly 25 minutes.

Testing. Following this training, participants were tested on how well they had learned the associations represented by the SP, ID, and SPID conditions. Participants were informed that, during training, configurations and identities of shapes were repeated. To assess their learning of these associations, each specific triple was presented, remaining on the screen until the participant responded. Their task was to select what type of repeated association was denoted by each triple: identities, positions, or both their identities and their positions.

MRI Scanning. Stimulus images were presented to participants through a head coil mirror that reflected the image of a 24inch MR compatible LCD display (BOLDScreen, Cambridge Research Systems LTD., UK) mounted at the head of the scanner bore. Each scanning session was comprised of, in order, six experimental condition runs, a high-resolution anatomical run, and a scene localizer run. All functional runs began and ended with 12s of fixation. The experimental runs were block design, with four stimuli, each repeated once, comprising a block of eight trials in total. Each block implemented one of nine conditions: SPID, SP, ID, NA, scenes (broken down into separate blocks for each scene category), objects, or scrambled. Each trial presented a stimulus with a fixation cross overlaid in the middle for 2s, to make a total duration of the block 16s. Runs were comprised of two blocks per condition, resulting in a total of four presentations of each stimulus image within a run. A given run contained 18 stimulus blocks (9 conditions x 2) interleaved with 10s fixation blocks; thus, the total duration of a run was seven minutes and thirty-two seconds. During the stimulus blocks, the participant’s task was to press a button when the fixation cross turned from green to red, which occurred twice per block.

The scene localizer was also block design, alternating scene blocks (n = 6) with object blocks (n = 6), with fixation blocks interleaved between them. Each block contained sixteen stimuli (14 unique), presented for 1s each, for a total block duration of 16s; fixation blocks were 8s. For both scene and object blocks, participants performed a one-back task—two stimuli repeated per block.

Re-testing. Following the MRI scanning session, participants were again tested on how well they had learned the associations represented by the SP, ID, and SPID conditions using methods identical to those used in the pre-scan test. Note that this re-test was incorporated into our experimental protocol only for participants 6–26, of whom ten are included in our fMRI analyses. For these participants, when correlating the BOLD response with behaviorally-assessed learning, we used the average of the test and the re-test as the behavioral measure.

### fMRI data acquisition

MRI data was collected on a 3T Siemens Verio MR scanner at the Scientific Imaging & Brain Research Center at Carnegie Mellon University using a 32-channel head coil. Functional images were acquired using a T2*-weighted echoplanar imaging pulse sequence (31 slices aligned to the AC/PC, in-plane resolution 2mm x 2mm, 3mm slice thickness, no gap, TR = 2000ms, TE = 29ms, flip angle = 79°, GRAPPA = 2, matrix size 96x96, field of view 192mm, reference lines = 48, descending acquisition). Number of acquisitions per run was 224 for the main experiment, and 152 for the scene localizer. High-resolution anatomical scans were acquired for each participant using a T1-weighted MPRAGE sequence (1mm x 1mm x 1mm, 176 sagittal slices, TR = 2.3s, TE = 1.97ms, flip angle = 9°, GRAPPA = 2, field of view = 256).

### fMRI analysis

Due to participant drowsiness and movement during the later course of the experiment, as well as adaptation to the stimuli, only fMRI data from the first two association runs were analyzed. For a more detailed description of why only run 1 and run 2 were included in the analysis, please refer to the S1 Supporting Methods.

Preprocessing. Functional data was analyzed using SPM8 (http://www.fil.ion.ucl.ac.uk/spm/). All data were realigned to correct for minor head motion by registering all images to the mean image and the anatomical image was co-registered with the functional images. For the ROI analyses, functional data from the experimental conditions were subjected to no additional preprocessing steps. Thus, all ROI analyses were only preprocessed for correcting for motion and did not have additional smoothing. For the group average of the whole brain analysis, the association data were normalized to the MNI template for averaging purposes, and smoothed with using an isotropic Gaussian kernel 4mm FWHM. Finally, the scene localizer fMRI data were smoothed using an isotropic Gaussian kernel (FWHM = 6mm).

General Linear Model. fMRI data were analyzed in a block design paradigm using a canonical hemodynamic response function. Each event was modeled within a 16s time window, and a high pass filter using 128s was implemented. The six output parameters from realignment were used as nuisance regressors within the model. The general linear model incorporated a robust weighted least squares (rWLS) algorithm [47] which yields estimated the noise covariates and temporal auto-correlation for later use as covariates within the design matrix. The association design modeled nine conditions: SPID, SP, ID, NA, scene category (3), objects, and scrambled. The scene localizer design modeled two conditions: scenes and objects. For the whole brain analysis in the group average the contrasts were passed to a second-level random effects analysis that consisted of testing the contrast against zero using a voxel-wise single-sample *t*-test. All group maps presented are whole brain analysis using an FDR correction of *q* < .05, minimum cluster size k = 10. For visualization purposes group average maps were rendered onto 3D inflated brains using the CARET software [48].

Region of interest (ROI) analyses. All ROI analyses were performed at the individual level using the MarsBaR toolbox (http://marsbar.sourceforge.net/index.html) and analyzed within native space. Data from the contrast of scenes versus objects in the separate scene localizer were used to define scene-selective regions within the PPA, RSC, and OPA for each individual. Typically, a threshold of FWE *p* < .01 was used to define the set of voxels, and there were no overlapping voxels across ROIs. For the anterior and posterior analysis of the PPA region, the PPA ROI was defined by first anatomically labeling the parahippocampal cortex (PHC) in each individual [49,50]. Using the voxel coordinates from the y-axis, the PHC was then divided in four equal (in y-domain) sections. These subregions were then functionally masked to only include voxels that were significantly active in the scene versus object contrast of the localizer. However, scene related activity within the parahippocampal/lingual region extended beyond the posterior border of the parahippocampal cortex and into the lingual gyrus. We included these more posterior voxels as the most posterior section, giving us a total of five sections of the parahippocampal place area from anterior to posterior regions. Average number of voxels across the subregions was 127 in the LH, and 141 in the RH. There were significant differences in the number of voxels across the subregions (LH: *F*(4,56) = 8.89, *p* < 0.000012; RH: *F*(4,56) = 19,85, *p* < .0000001). These differences, however, arise from middle subregions containing more voxels than the end subregions. Critically, in the LH the most anterior and most posterior regions were not significantly different in number of voxels (planned comparisons; *p* > .55), in the RH the anterior region had slightly more voxels than the most posterior region (*p* < .05). These ROIs were then applied in the analyses, paired *t*-tests and repeated measures ANOVAs, of the association data to extract weighted parameter estimates (i.e., beta values) averaged across all voxels for each condition compared with the baseline or the NA condition.

In the cross correlation analysis, unthresholded *t*-values from each of the voxels of a specific ROI were extracted from the contrasts of interest. These *t*-values were then cross-correlated on a voxel-by-voxel level within each ROI across the different contrasts as specified in the results section (e.g., Scenes vs. baseline correlated with SPID vs. NA). *R*-values for each individual contrast comparison, for each ROI, were then Fisher corrected to perform additional paired *t*-tests and repeated measures ANOVAs.

## Supporting Information

S1 Fig Posterior to Anterior gradient of processing spatial associations to identity associations in the right hemisphere. (JPG)

S1 File Supporting Methods. Supplemental information regarding participants and data selection.
(PDF)

S1 Table Significant (FDR q < .05, k = 10) clusters of overlapping activity in processing associations (SPID vs. NA) and scenes (scenes vs. objects & scrambled). PPA: parahippocampal/lingual region; OPA: occipital place area; RSC: retrosplenial complex; LH: left hemisphere; RH: right hemisphere.
(JPG)
