# Supplementary figures and images for: Associative Processing Is Inherent in Scene Perception
Source: PLoS One. 2015 Jun 12;10(6):e0128840. doi: 10.1371/journal.pone.0128840 (PMC4467091; doi:10.1371/journal.pone.0128840)

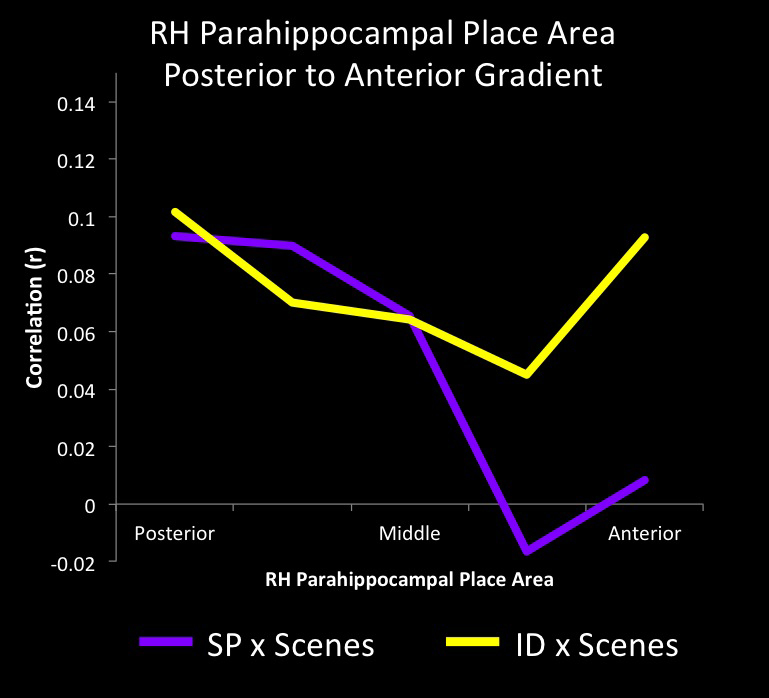

Supplement: S1 Fig — (JPG) [file pone.0128840.s001.jpg]

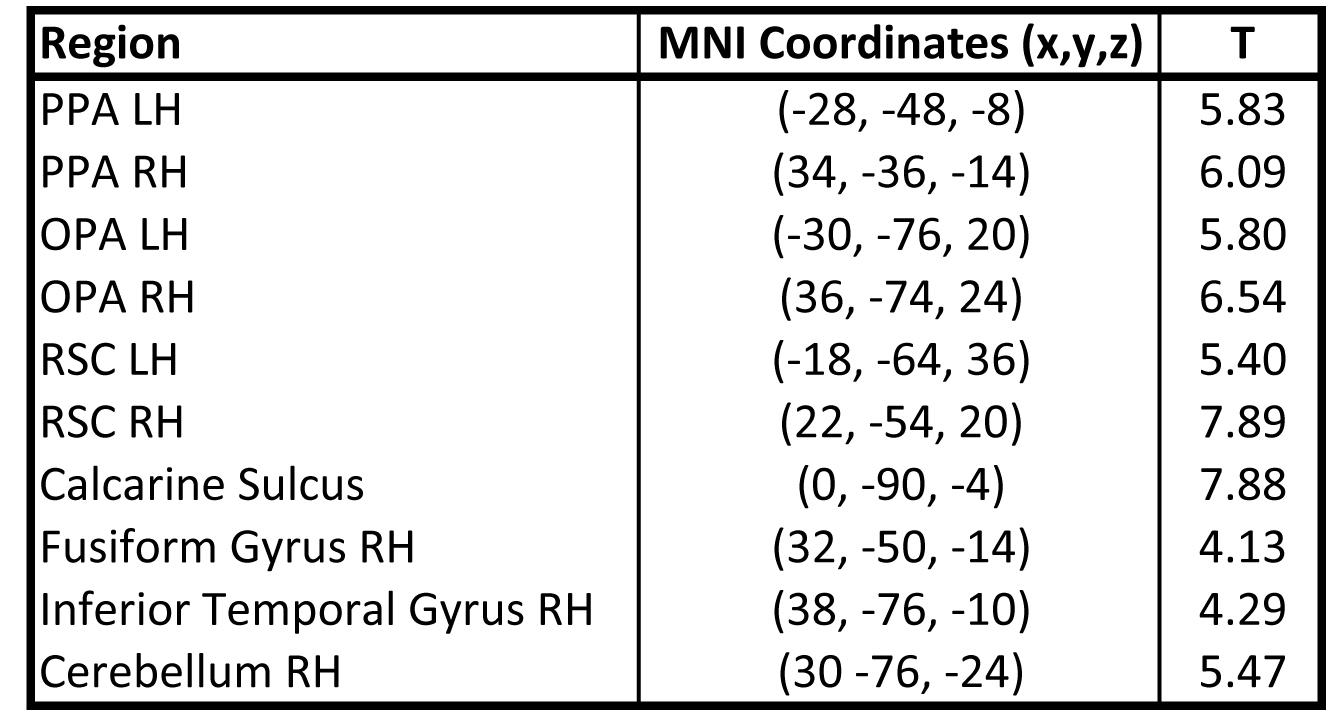

Supplement: S1 Table — PPA: parahippocampal/lingual region; OPA: occipital place area; RSC: retrosplenial complex; LH: left hemisphere; RH: right hemisphere. (JPG) [file pone.0128840.s003.jpg]
