# Supplementary material for: Associative Processing Is Inherent in Scene Perception
Source: PLoS One. 2015 Jun 12;10(6):e0128840. doi: 10.1371/journal.pone.0128840 (PMC4467091; doi:10.1371/journal.pone.0128840)
Supplement: S1 File — Supplemental information regarding participants and data selection. (PDF) [file pone.0128840.s002.pdf]

Supporting Information:

Associative processing is inherent in scene perception

Elissa M. Aminoff & Michael J. Tarr

Supporting Methods:

Participants

Twenty-six people participated in this study, however eleven participants were excluded from the analysis for: extreme movement, > 3mm (3); dozing off, as implied by missed responses and discussions with the participant (3); movement and dozing off (3); irregular large arachnoid cyst near ventral temporal/occipital cortex (1); irregular BOLD activity map yielded from the scene versus objects and scrambled contrast (1). In contrast, the fifteen participants with movement < 3mm or with smooth, cumulative linear motion between 3mm and 4mm were included in further analyses. However, as discussed next, for principled reasons based on cumulative motion and missed trial rates as the experiment progressed, only the first two runs were included in our statistical analyses.

Data selection

In our analyses, only the initial two of the six runs were included. This selection was based on the fact that there were a very limited number of stimuli – only 4 unique stimuli in each condition. This small number of stimuli was based on the difficulty of implicitly learning complex associative stimuli. Given only 4 stimuli per condition, specific stimuli were repeated many times: twice per block, four times per run, twenty-four times during the experiment. This repetition, along with a very low-engagement task in which the participant monitored for a fixation cross color change (twice per block), meant that participants may have been disengaged during the latter portions of the experiment. Many participants reported becoming drowsy and falling asleep (verified by the experimenter), others tended to fidget as the experiment progressed. At the same time, stimulus repetition reduced the BOLD signal over the course of the experiment due to adaption, which we elaborate on below. In light of these issues, we considered only the first two runs which showed the lowest levels amount of participant movement, missed trials, and adaptation. In part, guided by our behavioral data, we chose to use only the first two runs of the study in that these runs were the only ones in which all the participants performed at high levels, missing less than 10% of the task trials (see range values in Row 1, Table S2). Reinforcing our assumption that Runs 1 and 2 reflect better data quality relative to Runs 3-6, the cumulative movement across Runs 1 and 2 was relatively small for all participants, whereas across all six runs, some participants showed unsatisfactory levels of movement. As such, inclusion of the first two runs for further analyses was based on a principled model of participant behavior in which increasing fatigue and distraction led to suboptimal levels of both missed responses and subject movement. In particular, we did not perform statistical analyses on different combinations of runs, which would have led to bias in our results. Tables S2 and S3 present a quantitative description of these characteristics across runs.

Table S2: Number of trials that the participants missed responding when the fixation cross changed color.

Missed Trials	Run 1	Run 2	Run 3	Run 4	Run 5	Run 6
Range	[0 - 3]	[0 - 3]	[0 - 7]	[0 - 7]	[0 - 8]	[0 - 6]
Average	0.66	0.866	1.4	1.07	1.4	1.27
Stdev	1.11	1.12	1.88	2.12	2.3	2.12

Table S3: Maximum cumulative movement across runs in the MRI scanner in both the translation and rotation directions.

Max Cumulative Movement	Run 1		Run 2		Run 3		Run 4		Run 5		Run 6	
Type	Translation (mm)	Rotation (deg)	Translation (mm)	Rotation (deg)	Translation (mm)	Rotation (deg)	Translation (mm)	Rotation (deg)	Translation (mm)	Rotation (deg)	Translation (mm)	Rotation (deg)
Range	[0.2 - 1.1]	[0.2 - 1.3]	[0.4 - 1.3]	[0.3 - 1.7]	[0.6 - 2.3]	[0.6 - 2.3]	[0.7 - 2.7]	[0.7 - 2.3]	[0.7 - 4.0]	[0.7 - 2.3]	[0.7 - 4.1]	[0.7 - 2.3]
Avg	0.58	0.51	0.92	0.89	1.33	1.14	1.56	1.32	1.95	1.39	2.11	1.41
Stdev	0.27	0.28	0.25	0.41	0.54	0.44	0.53	0.45	0.82	0.45	0.92	0.45

With regard to adaptation effects, the few number of stimuli led to reduced signal as the duration of the experiment increased. This was true for the associative shape conditions, as well as for the scene conditions. The data presented with respect to any adaptation effects across Runs 1-6 should be considered with caution in that this analysis includes data, as illustrated above, with high levels of movement and distraction/boredom. Note also that this across-run analysis was implemented in response to reviewer comments long after all other analyses in this report were conducted. This is illustrated in Figure S2: in both the associative processing contrast (the average of SPID, SP, and ID versus NA) and the scene processing contrast (Scenes versus Objects and Scrambled) the magnitude of the effect decreased. The reduction was not significantly different between these contrasts, except in the LH OPA where the reduction was more pronounced in the associative processing condition. As discussed in our Results section, the OPA was negatively correlated with learning, and it may be that this region is only engaged in the very early processing of associations and drops off once an association has been established. The fact that we observe a comparable reduction in signal for associative processing and scene processing suggests that this is an artifact of adaptation.

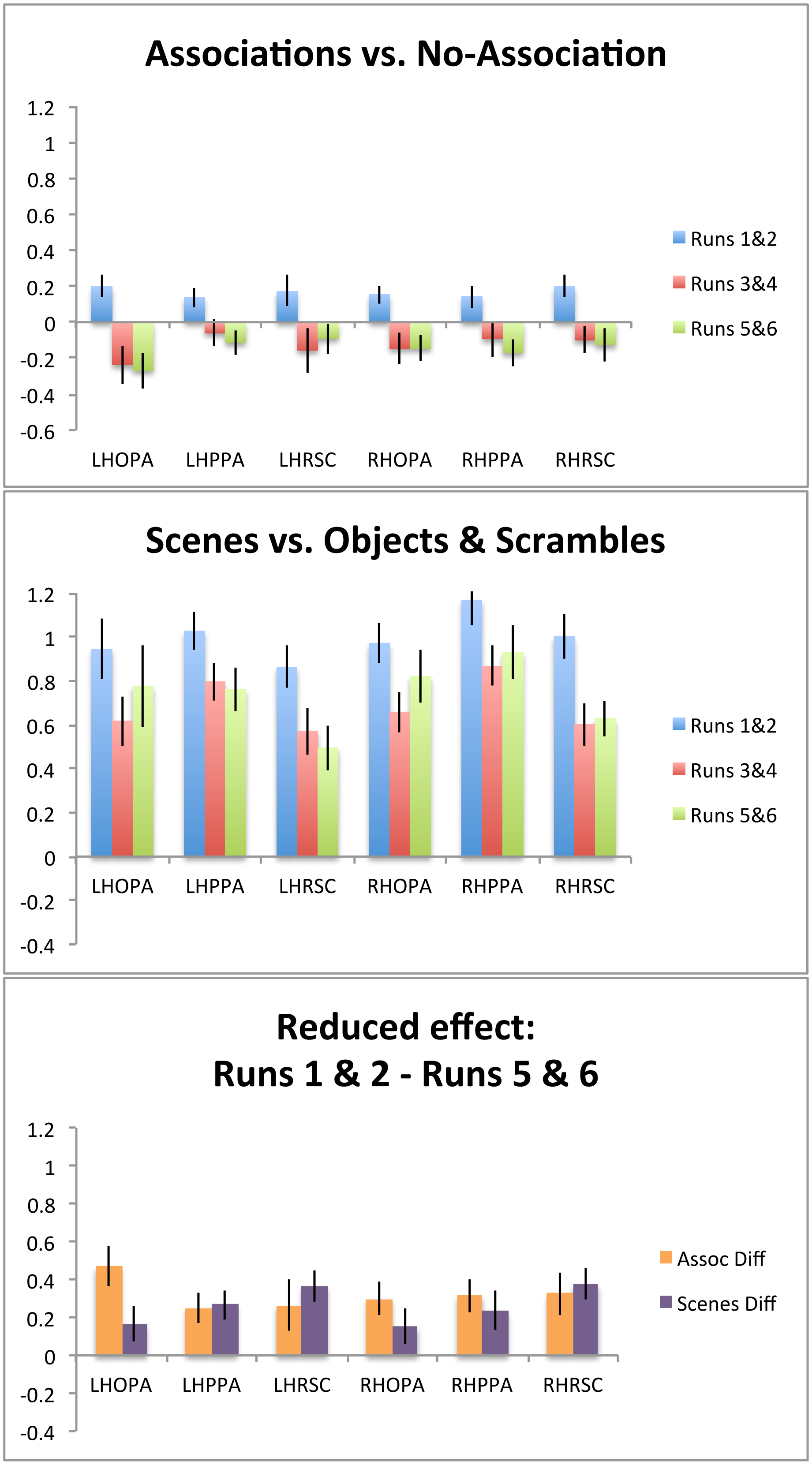

Figure S2: Three graphs examining the adaption in stimuli across runs. Top: The difference in activity for the associative shapes (SPID, SP, and ID collapsed) and the NA shapes for runs 1 and 2 in blue (the

data used in the main text), runs 3 and 4 in red, and runs 5 and 6 in green. **Middle:** The difference in activity for the scenes compared with the weak contextual objects and the scrambled images for runs 1 and 2 in blue (the data used in the main text), runs 3 and 4 in red, and runs 5 and 6 in green. **Bottom:** The adaptation (i.e., the reduced differential activity) of runs 5 and 6 from runs 1 and 2 for associative processing in orange, and in scene processing in purple. Only in the LH OPA was there a significant difference.
